# Beyond Bone Grafts: Exploring the Efficacy of Alternative Regenerative Therapies

**DOI:** 10.7759/cureus.73745

**Published:** 2024-11-15

**Authors:** Tsvetalina Gerova-Vatsova

**Affiliations:** 1 Department of Periodontology and Dental Implantology, Medical University of Varna, Varna, BGR

**Keywords:** autogenous platelet-rich plasma, barrier membrane, enamel matrix derivative, guided tissue regeneration, periodontology, platelet-rich plasma (prp), regenerative therapy, vertical bone defects

## Abstract

Context

A vast body of published literature examines and evaluates the properties of bone restorative materials in combination with other biomaterials or as stand-alone applications. If we exclude the studies investigating the effectiveness of regenerative therapy with enamel matrix derivative (EMD), in all other cases, bone regenerative materials are placed on a "pedestal." Therefore, the study we have initiated covers methods whose protocol does not use bone-repair materials. The clinical and radiographic results obtained are compared to determine which of these methods is the most reliable. The most important goal we set out was to determine if periodontal regenerative therapy would be effective without the use of bone graft restorative materials.

Aim

This study aimed to investigate, analyze, and compare the outcomes of four groups of patients with vertical bone defects (infrabony defects (IBDs)) who were treated using regenerative methods without the involvement of bone repair materials.

Materials and methods

Forty-eight cases that fulfilled all participation criteria for the study were selected. The O'Leary plaque index (PI) and Ainamo and Bay gingival index (GI) were assessed at the reassessment visit after the Hygiene Phase, the current periodontal status was recorded, and at least one IBD was identified. Cone beam computed tomography (CBCT) was ordered, and the size of each defect was measured by three parameters. In this study, all IBDs were randomly allocated to four groups. The first category encompasses IBDs, wherein regenerative therapy utilizing autogenous, platelet-rich plasma (PRP) is implemented. The second group comprises IBDs, which undergo regenerative therapy utilizing EMD. The third category encompasses IBDs in which guided tissue regeneration (GTR) is conducted using solely a barrier membrane. The fourth group encompasses IBDs, wherein GTR utilizing a barrier membrane and PRP took place. Six months after regenerative therapy, regardless of which of the four methods was used, all patients were reassessed clinically by CBCT. Statistical methods were used to evaluate, analyze, and compare the results in the four groups.

Results

A statistically significant decrease in the "probing pocket depth" indication, a statistically significant clinical attachment level gain, and a statistically significant decrease in the CBCT indicators "A" and "B" were observed in all four groups of patients under study. When it comes to the CBCT indicator "C," the results for each of the four groups of patients under study fall somewhere between statistical significance and non-significant.

Conclusions

Regardless of the regenerative therapy technique used, all patients under examination showed improvements in imaging and clinical markers. The four patient groups' results did not differ in any way that was statistically significant.

## Introduction

Periodontitis is among the most prevalent disease in the oral cavity. The disease is essentially infectious and inevitably leads to inflammation and destruction affecting all components of the periodontium [1,2]. Therefore, in periodontal treatment, two fundamental stages stand out today. The first stage is responsible for controlling and eliminating the inflammatory process (hygienic phase). The second stage of treatment is related to the restoration of the periodontal components that have been destroyed (corrective phase) [3].

The first areas to be affected in the development of periodontal disease are the distal interdental areas. These locations significantly hinder patients' ability to maintain personal dental hygiene. Unless timely measures are taken to motivate and educate patients to clean these areas more diligently and with better quality, the development of a periodontal problem is inevitable [4].

Goldman and Cohen classified defects as supraosseous and infraosseous [5]. Infrabony defects (IBDs) develop as a result of vertical bone resorption and are characterized by their base positioned apically to the residual alveolar ridge [5,6].

Diagnosis of these defects is done clinically and radiographically. Due to its significant advantages over conventional 2D radiographs, today, cone beam computed tomography (CBCT) is the preferred method for making an accurate diagnosis, as well as for assessing the size and morphology of the bone defect [7,8]. For qualitative and quantitative bone evaluation, CBCT along with bone histology and histomorphometry are the methods of choice [9,10,11].

It is an undeniable fact that IBDs can be treated. The reason for this lies in the remarkable possibilities of regenerative methods, restoring both the soft and hard components of the periodontium [12].

Although periodontal regenerative therapy has good predictive values, it still poses a huge challenge for dentists. It is no coincidence that new, more sophisticated biomaterials are being developed every day in order to improve outcome data [13,14,15,16,17]. A vast amount of the published literature investigates and evaluates the properties of bone regenerative materials in combination with other biomaterials or as stand-alone applications. If we exclude the studies investigating the effectiveness of regenerative therapy with self-administration of enamel matrix derivatives (EMDs), then in all other cases, bone regenerative materials are placed on a "pedestal" [18,19,20].

Therefore, the study we have initiated covers methods whose protocol does not use bone repair material. Both clinical and CBCT parameters are being investigated to achieve greater precision in the conclusions. The results are compared to determine which of these methods is most reliable. However, the most important goal we set out was to establish whether periodontal regenerative therapy would be an effective method without the use of a bone graft.

## Materials and methods

**Figure 1:**
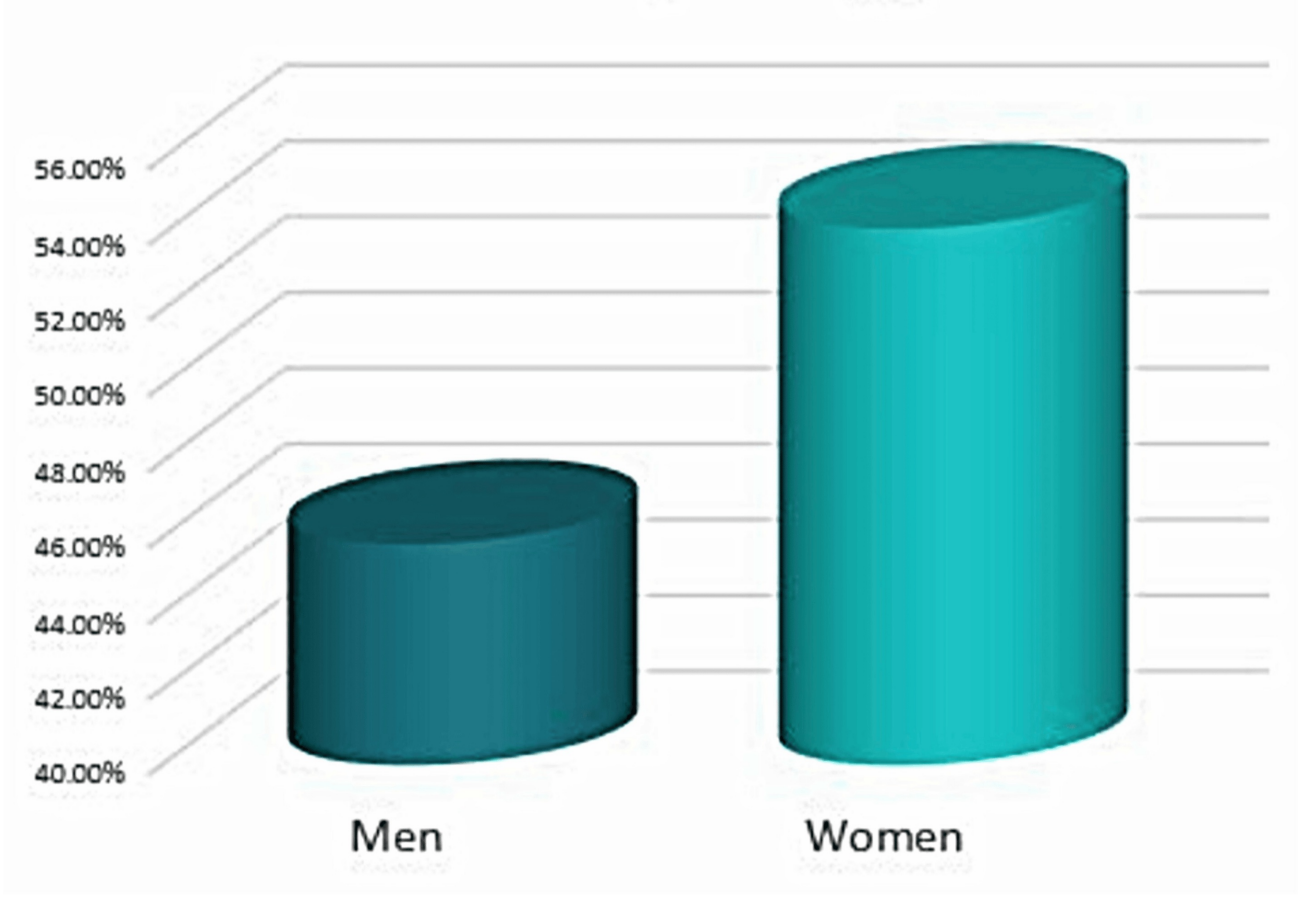
Allocation of the patients by gender

**Figure 2:**
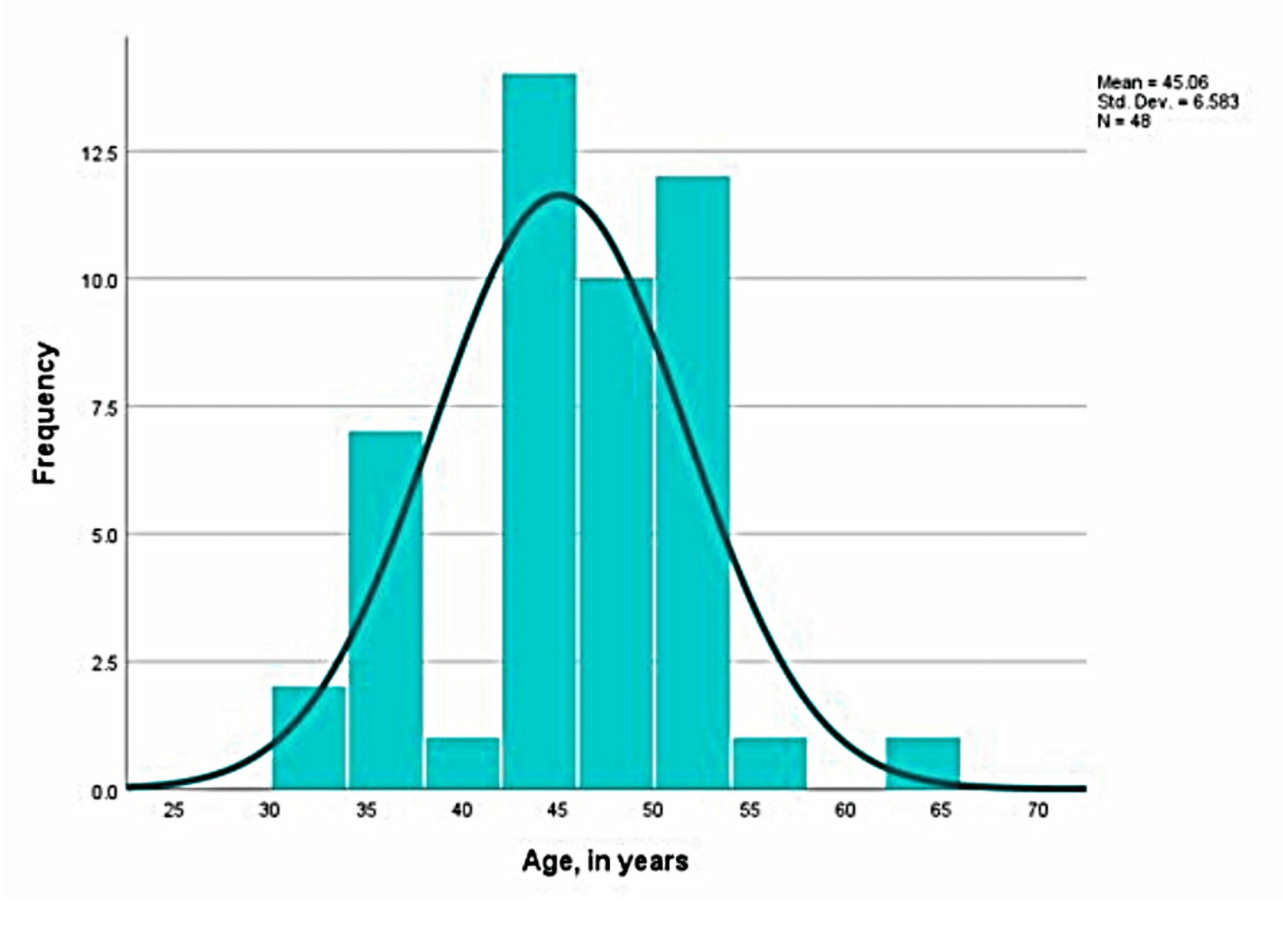
Allocation of the patients by age

The University Medical and Dental Center, part of the Medical University Varna, Faculty of Dental Medicine, was utilized for this research from August 2022 until July 2023. Forty-eight cases from a total of 30 male and female patients, aged 31 to 63 years (Figures 1, 2), who fulfilled all participation criteria for the study were selected - high degree of motivation to maintain excellent oral hygiene on the part of the patient, completed and signed informed consent and questionnaire on current health status excluding existing systemic diseases. A standard age range between 18 and 65 years was introduced.

**Figure 3:**
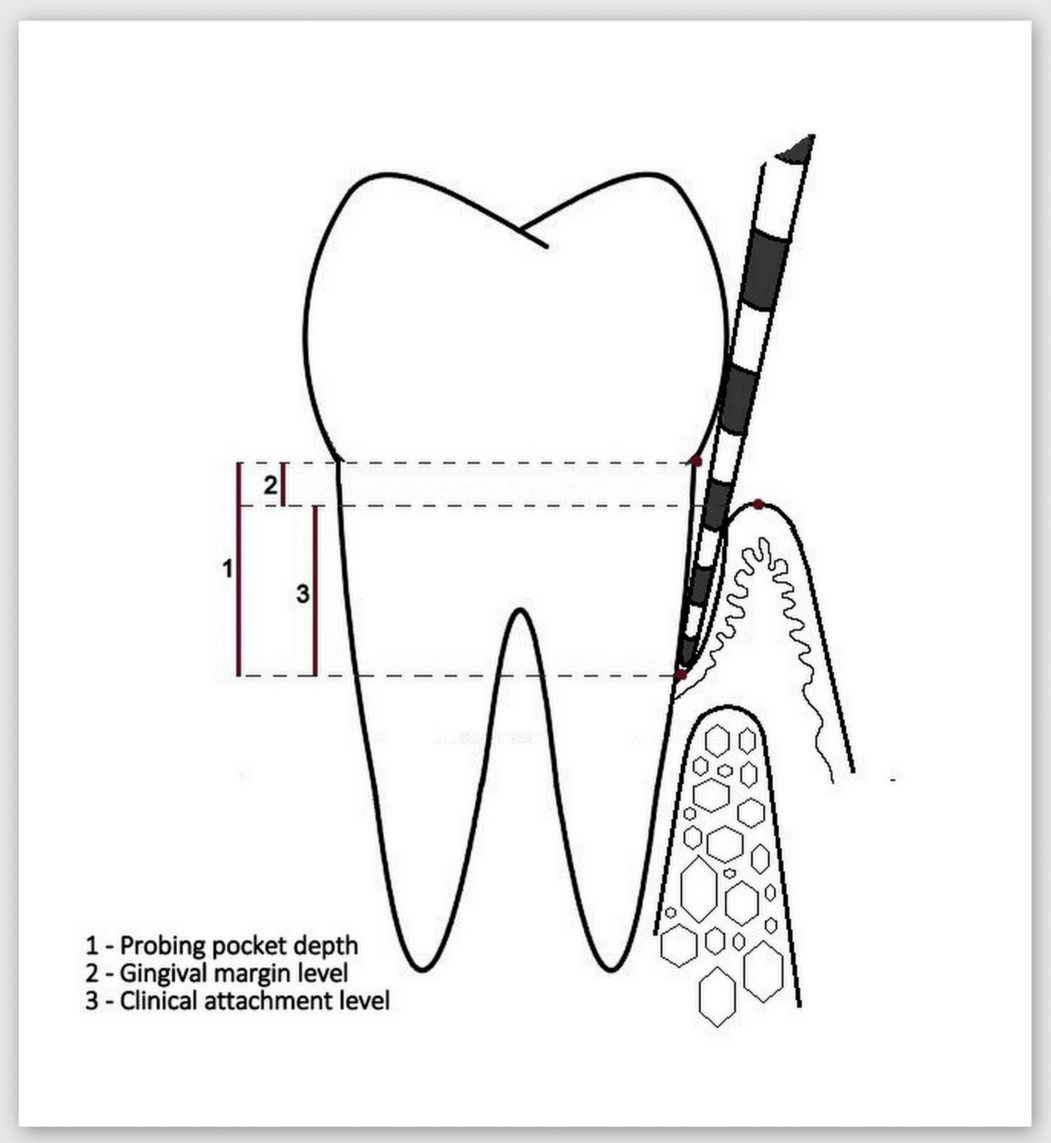
Examined clinical parameters under this study Image credits: Author Gerova-Vatsova T

**Figure 4:**
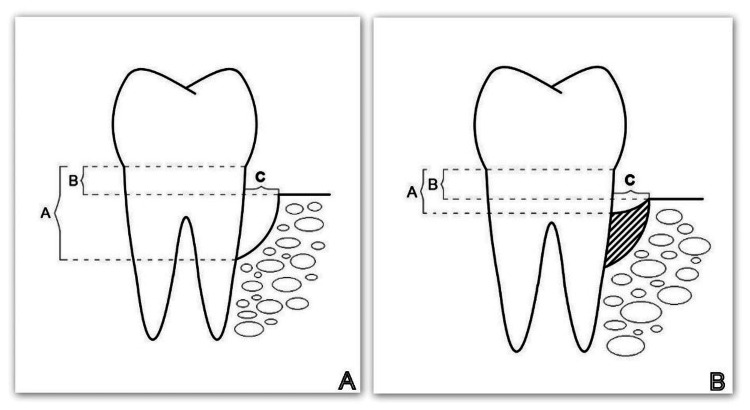
Examined radiographic parameters (A, B, and C) A: CBCT parameters studied before the regenerative therapy; B: CBCT parameters studied six months after the regenerative therapy. Parameter A: the distance from the cemento-enamel junction (CEJ) to the bottom of the bone defect. Parameter B: the distance from the CEJ to the apex of the bone defect. Parameter C: the width of the defect. CBCT: cone beam computed tomography Image credits: Author Gerova-Vatsova T

Between six and eight weeks after completion of the hygiene phase, a reassessment visit was scheduled for each study patient. The O'Leary plaque index (PI) and Ainamo and Bay gingival index (GI) were assessed, and the current periodontal status was recorded (clinical parameters examined were probing pocket depth (PPD), gingival margin level (GML), and clinical attachment level (CAL) (Figure 3)), and at least one IBD was identified. In all patients found to have an infrabony defect indicated for surgical treatment, a CBCT scan was ordered and three parameters (described in Figure 4A) assessing the size and morphology of the defect were measured.

After completion of the evaluation and analysis of the initial clinical and radiological data, a date for the surgical intervention was scheduled.

**Figure 5:**
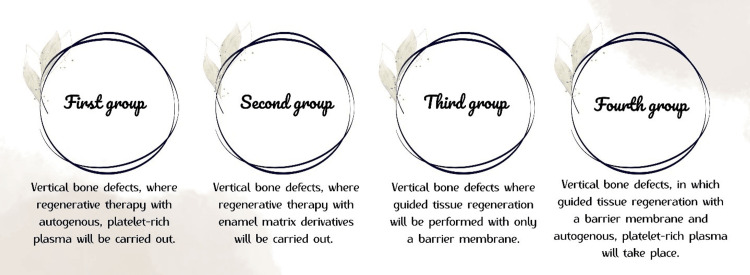
The four study groups in the study Image credits: Author Gerova-Vatsova T

In this study, all IBDs were randomly allocated to four groups. The first category encompasses IBDs, wherein regenerative therapy utilizing autogenous, platelet-rich plasma (PRP) is implemented. The second group comprises IBDs, which undergo regenerative therapy utilizing EMD. The third category encompasses IBDs in which guided tissue regeneration (GTR) is conducted using a collagen barrier membrane (Botiss Jason membrane, Berlin, Germany). The fourth group encompasses IBDs, wherein GTR utilizing a collagen barrier membrane (Botiss Jason membrane, Berlin, Germany) and PRP took place (Figure 5).

At six months after the regenerative therapy, regardless of which of the four methods were used, clinical and CBCT parameters were measured again in all patients (Figure 4B). Statistical methods were used to evaluate, analyze, and compare the results in the four groups.

## Results

Initially, this study must exhibit and compare the PI and GI acquired from the participants across all four groups to ascertain the mutual validity of our results.

**Figure 6:**
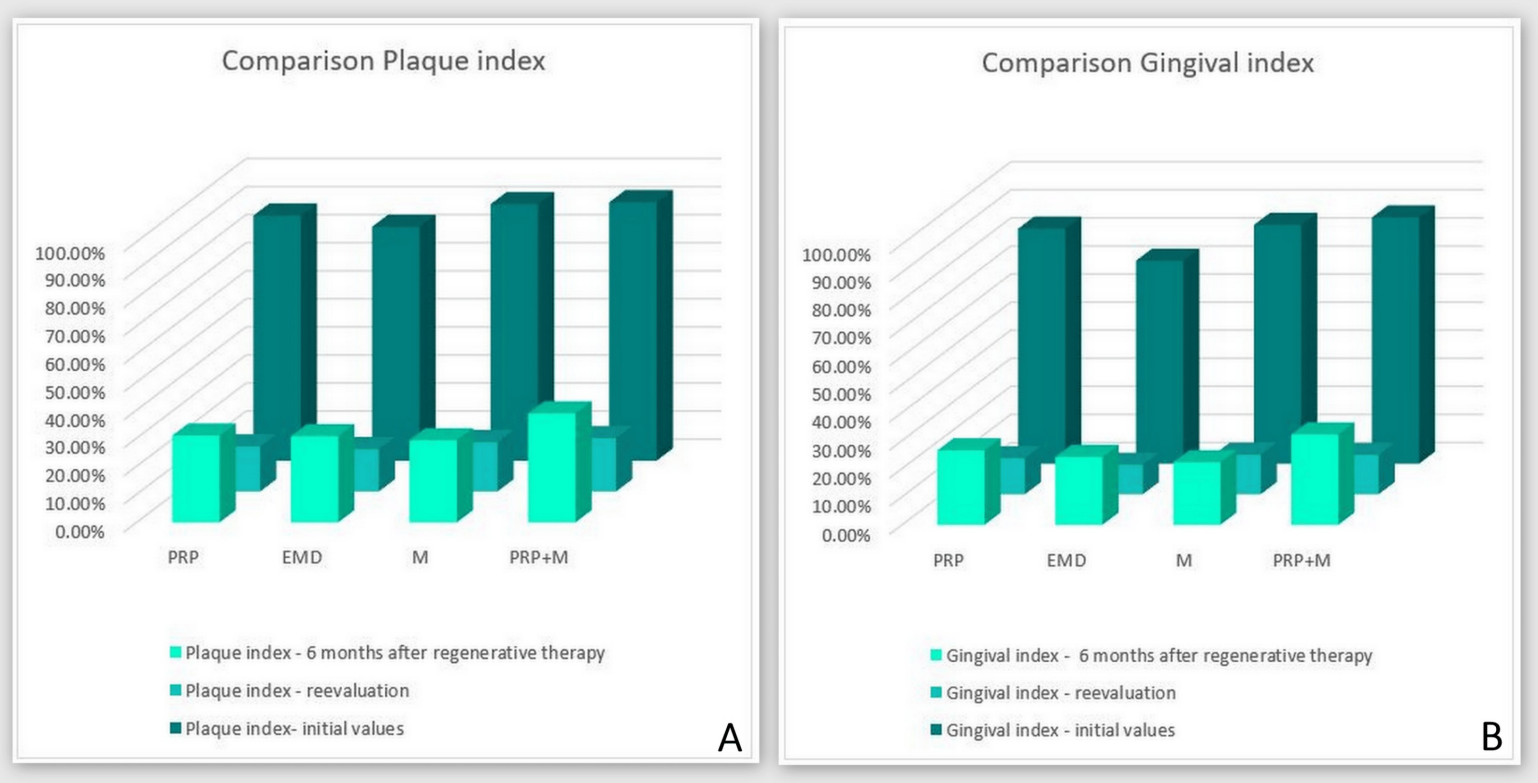
PI and GI acquired from the participants across all four groups PI: O'Leary plaque index, GI: Ainamo and Bay gingival index, PRP: platelet-rich plasma, EMD: enamel matrix derivative, M: barrier membrane, PRP+M: autogenous platelet-rich plasma and barrier membrane

Figure 6 distinctly illustrates the mean percentages of the PI and GI for patients across each group during three reporting time frames: 1) immediately before the beginning of the periodontal treatment; 2) reassessment following the hygiene phase; and 3) six months subsequent to the execution of regenerative therapy. A clear link exists between PI and GI across the three time frames of periodontal therapy among the subjects.

It is evident that the plaque and gingival indices before the initiation of periodontal therapy were markedly elevated in each participant, indicating that all subjects in the study commenced with inadequate personal oral hygiene and a significant percentage of gingival inflammation. During the reassessment phase, there was a notable reduction in the values for both indices across all four groups, indicating the substantial cooperation of every individual during periodontal therapy. At the six-month post-regenerative therapy phase, there was an elevation in both the PI and GI scores, which may be attributed to a decline in the patients' individual oral hygiene practices. The PI values at stage 3 across the four groups were essentially equivalent, so permitting the exclusion of PI and GI as modifiers in the outcomes of any of the groups.

Probing pocket depth

**Figure 7:**
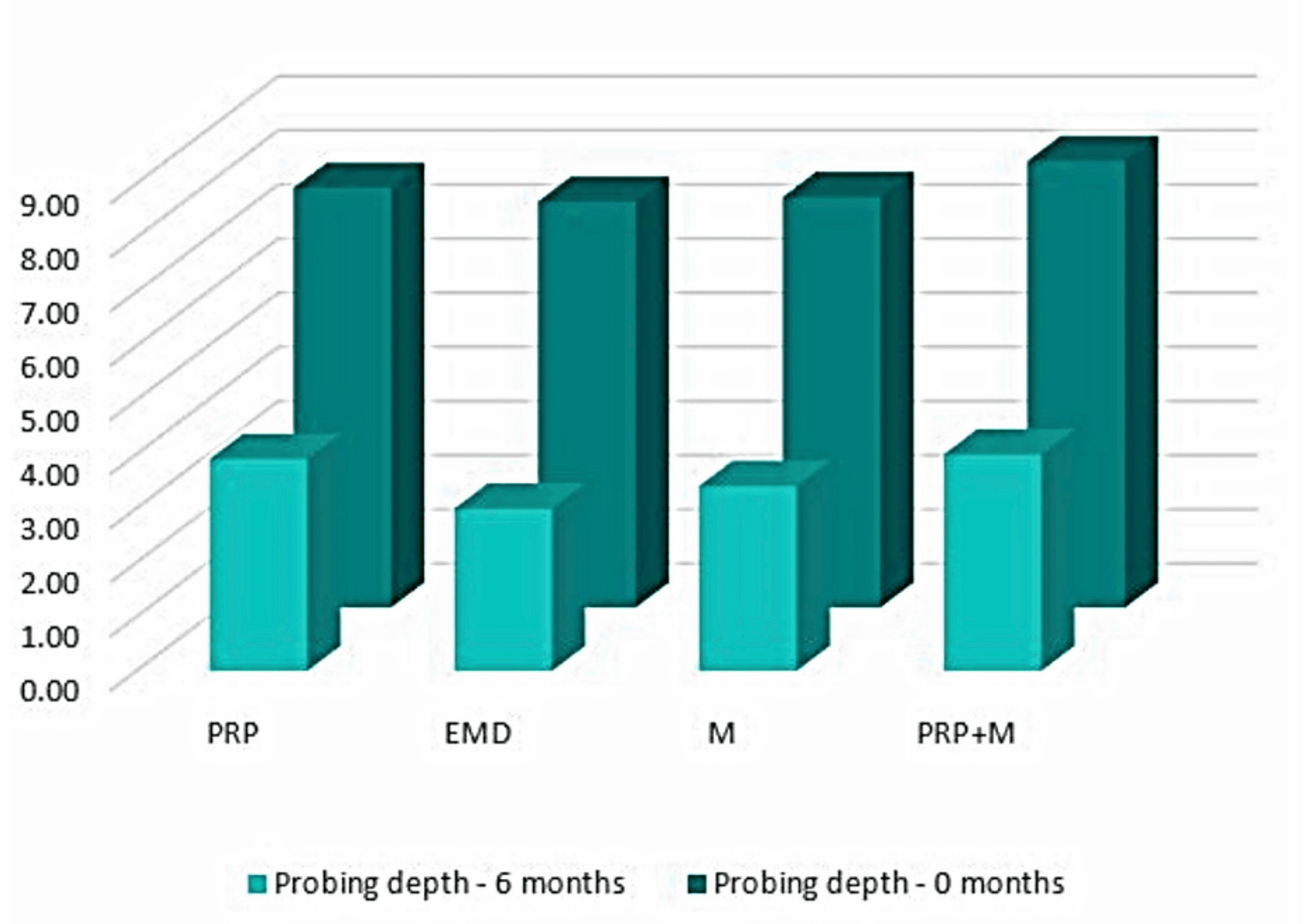
PPD comparison between 0 and six months PPD: probing pocket depth, PRP: platelet-rich plasma, EMD: enamel matrix derivative, M: barrier membrane, PRP+M: autogenous platelet-rich plasma and barrier membrane

Figure 7 illustrates that during the hygienic phase, the PPD parameter exhibited a notably elevated mean value across all four patient groups (first group = 7.75 mm; second group = 7.50 mm; third group = 7.58 mm; fourth group = 8.25 mm). In the six-month post-regenerative therapy, a notable decrease in PPD was seen relative to baseline measurements (first group = 3.92 mm; second group = 3.00 mm; third group = 3.42 mm; fourth group = 4.00 mm). The comprehensive results are displayed in Table 1.

**Table 1:** Comparison of the clinical and radiographic parameters at 0 and six months PRP: autogenous platelet-rich plasma, EMD: enamel matrix derivatives, M: barrier membrane, PRP+M: autogenous platelet-rich plasma and barrier membrane, PPD: probing pocket depth, GML: gingival margin level, CAL: clinical attachment level

Method	PPD - 0 months	GML - 0 months	CAL - 0 months	PPD - 6 months	GML - 6 months	CAL- 6 months	A - 0 months	B - 0 months	C - 0 months	A - 6 months	B - 6 months	C - 6 months
PRP	7.75	0.08	-7.67	3.92	0.17	-3.75	6.37	3.19	2.37	4.68	2.69	2.01
EMD	7.50	-0.08	-7.58	3.00	-0.58	-3.58	5.85	2.64	2.07	4.34	2.14	1.84
M	7.58	-0.33	-7.92	3.42	-0.67	-4.08	7.14	3.61	1.89	5.46	3.12	1.78
PRP+M	8.25	-0.58	-8.83	4.00	-0.33	-4.33	7.15	3.31	2.37	5.74	2.93	1.86

**Figure 8:**
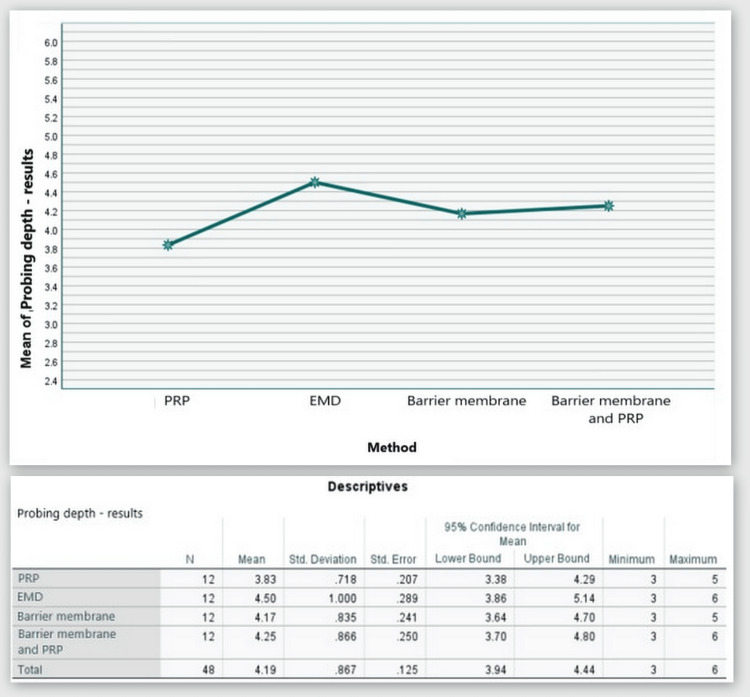
Statistical analysis results and graphics for the PPD PPD: probing pocket depth

Based on the statistical analyses, it was found that PPD was decreased by 3.83 mm on average in Group 1. In Group 2, it was decreased by 4.50 mm. In Group 3, it was decreased by 4.17 mm on average. In Group 4, it was decreased by 4.25 mm on average (Figure 8).

The ANOVA test demonstrated that the findings for PPD at six months post-regenerative therapy were not statistically significant among all four patient groups, F(3,44) = 1.228, p = 0.311 > 0.05 (Appendix A).

Gingival margin level

**Figure 9:**
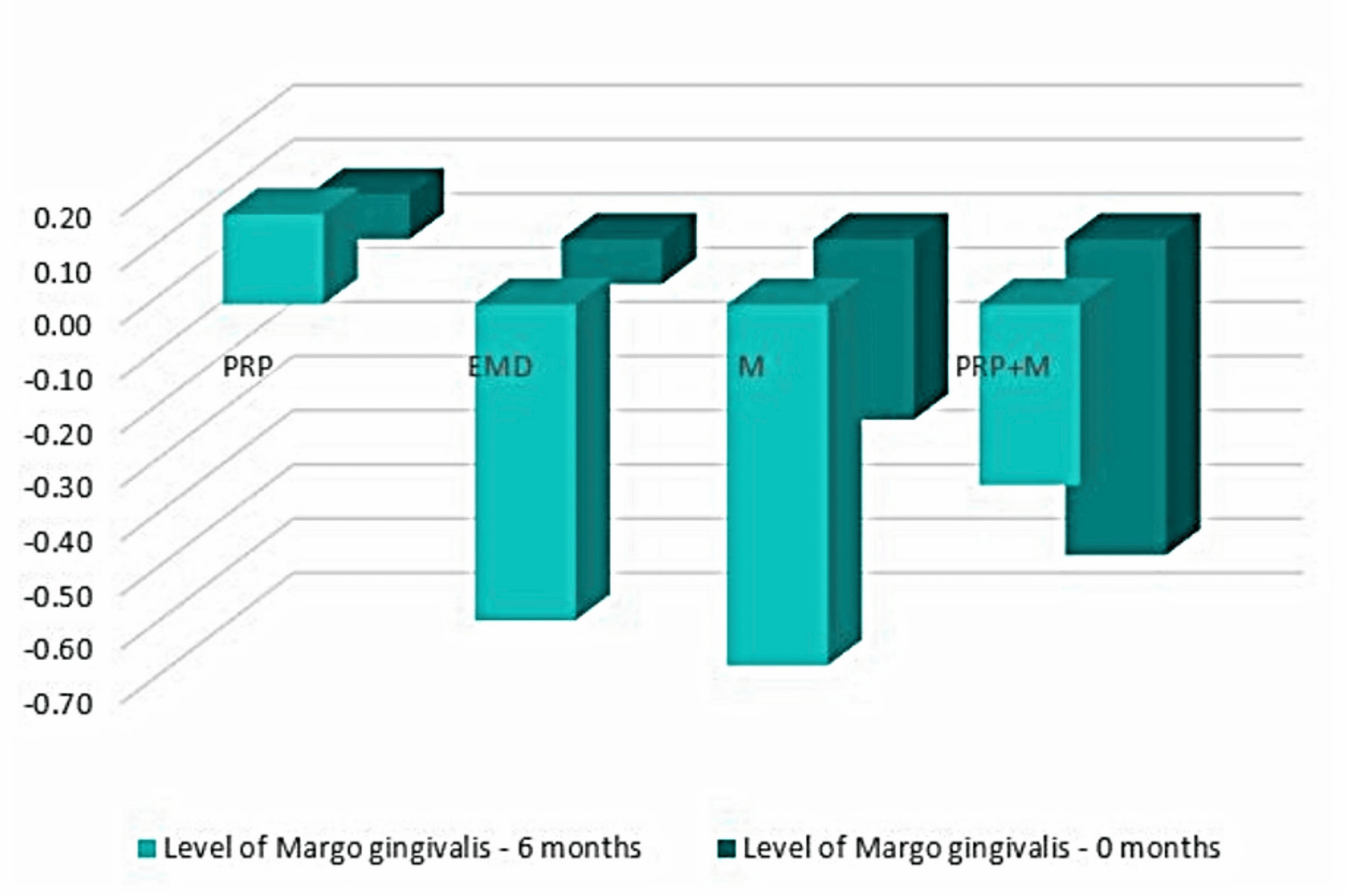
GML comparison at 0 and six months GML: gingival margin level, PRP: platelet-rich plasma, EMD: enamel matrix derivative, M: barrier membrane, PRP+M: autogenous platelet-rich plasma and barrier membrane

Figure 9 illustrates that throughout the hygienic phase, the mean values of the GML parameter were 0.08 mm for Group 1, -0.08 mm for Group 2, -0.33 mm for Group 3, and -0.58 mm for Group 4. At six months post-regenerative therapy, there was a relative retention of values compared to the baseline measurements (Group 1 = 0.17 mm; Group 2 = -0.58 mm; Group 3 = -0.67 mm; Group 4 = -0.33 mm). This indicates that clinically, there was virtually no apical or coronal migration of the gingival margin seen. The findings are elaborated upon in Table 1.

**Figure 10:**
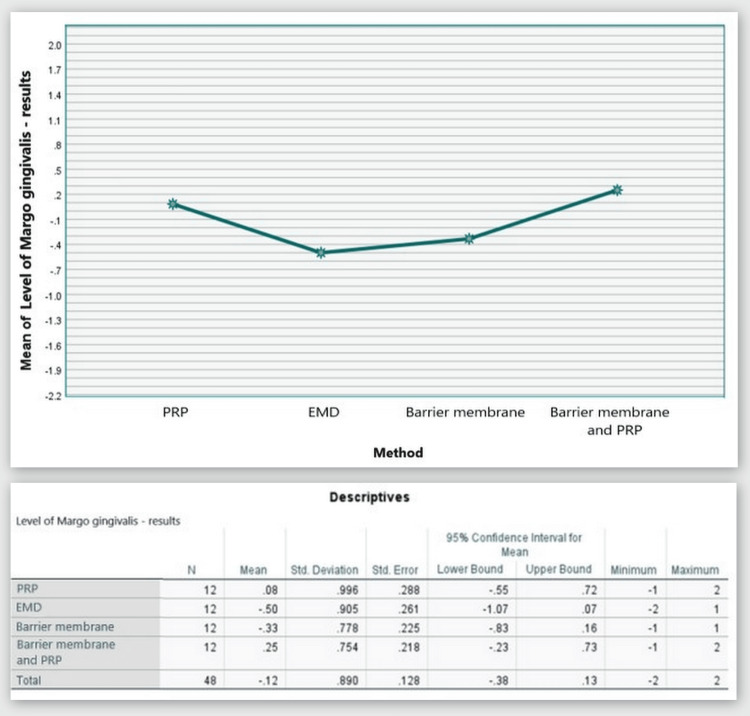
Statistical analysis results and graphics for GML GML: gingival margin level

Based on the statistical analyses, Groups 1 and 4 showed a shift of the gingival margin in the coronal direction by an average of 0.08 mm and 0.25 mm, respectively, while the other two groups showed a shift of the gingival margin in the apical direction by an average of 0.5 mm and 0.33 mm (Figure 10).

The ANOVA test demonstrated that the findings for GML at six months post-regenerative therapy were not statistically significant among all four groups: F(3, 44) = 1.973, p = 0.132 > 0.05 (Appendix B).

Clinical attachment level

**Figure 11:**
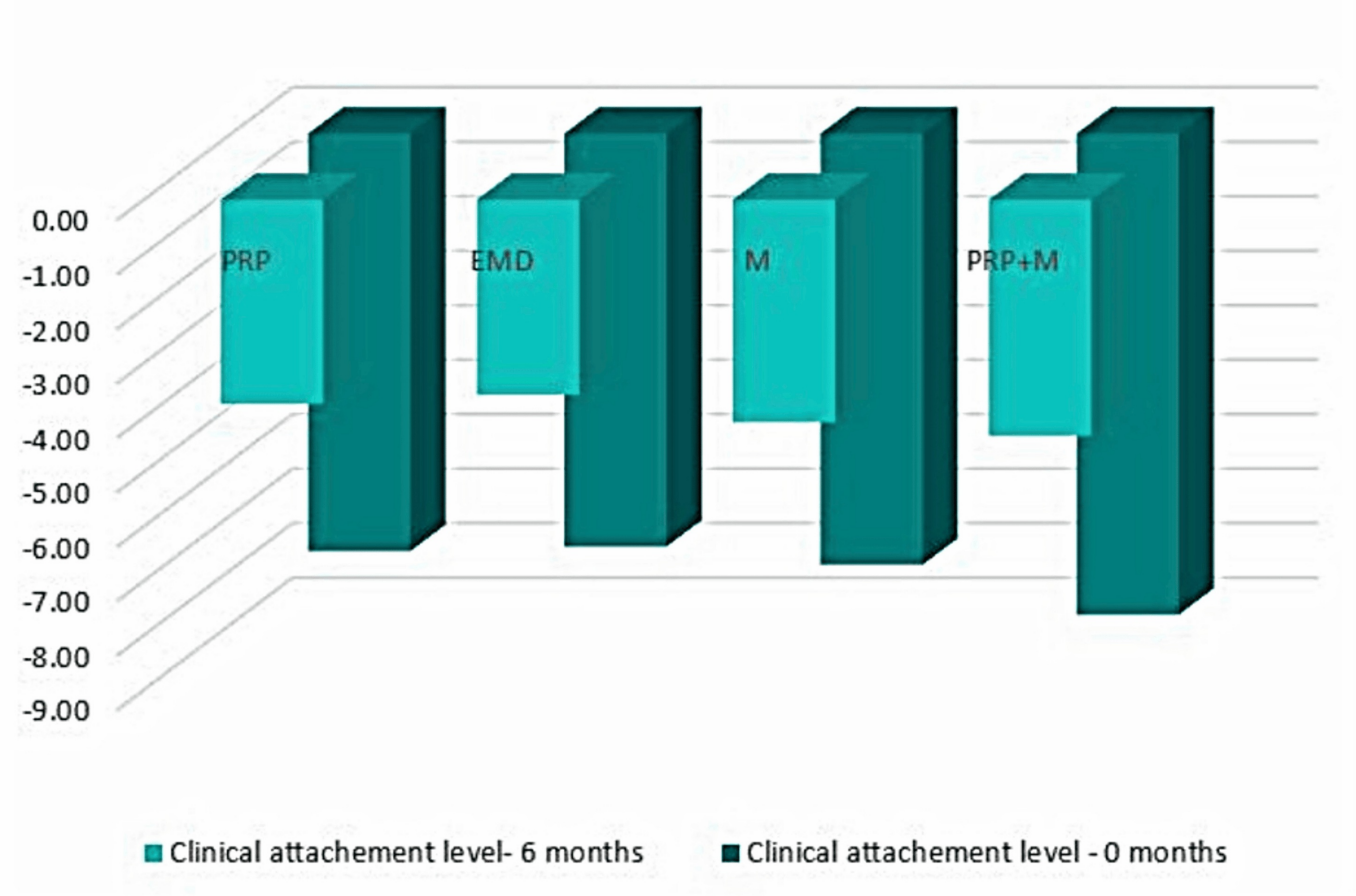
CAL comparison at 0 and six months CAL: clinical attachment level, PRP: platelet-rich plasma, EMD: enamel matrix derivative, M: barrier membrane, PRP+M: autogenous platelet-rich plasma and barrier membrane

Figure 11 shows that throughout the hygienic phase, Group 1 patients had a CAL measurement of 7.67 mm, Group 2 - 7.58 mm, Group 3 - 7.92 mm, and Group 4 - 8.83 mm. Six months after the regeneration therapy, all methods showed a significant reduction in these values, indicating CAL gain (Group 1, 3.75 mm; Group 2, 3.58 mm; Group 3, 4.08 mm; and Group 4, 4.33 mm on average).

**Figure 12:**
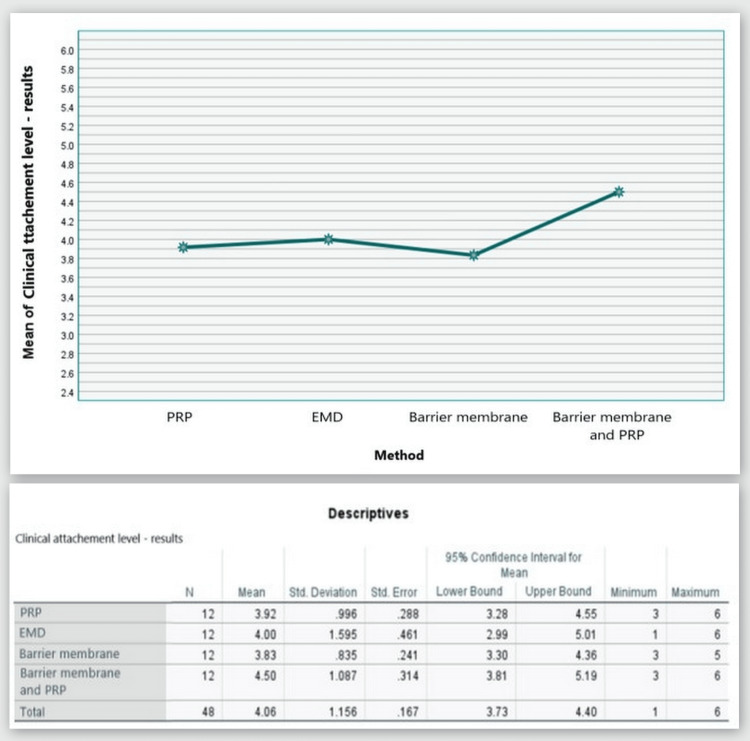
Statistical analysis results and graphics for the CAL CAL: clinical attachment level

As visible in Figure 12, CAL gain was observed in all four groups with mean values of 3.92, 4.00, 3.83, and 4.50 mm, respectively.

The ANOVA test demonstrated that the findings for CAL at six months post-regenerative therapy were not statistically significant among all four groups: F(3, 44) = 0.795, p = 0.503 > 0.05 (Appendix C).

А: the distance from the cementoenamel junction (CEJ) to the base of the bone defect (by CBCT)

**Figure 13:**
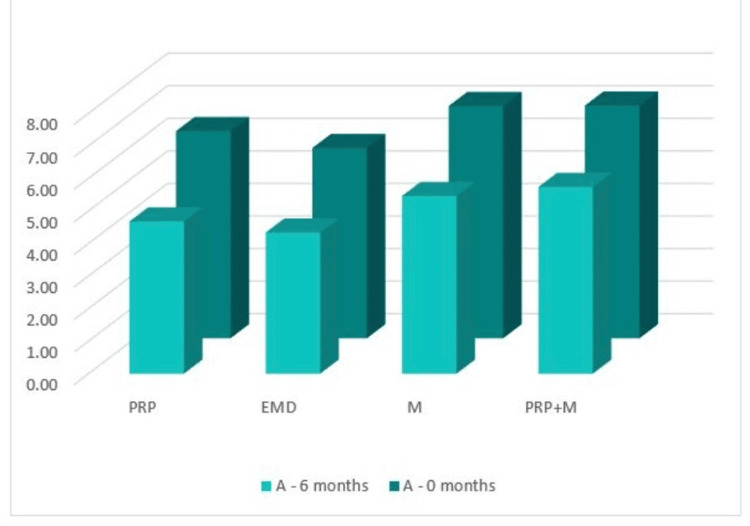
Parameter A comparison at 0 and six months PRP: platelet-rich plasma, EMD: enamel matrix derivative, M: barrier membrane, PRP+M: autogenous platelet-rich plasma and barrier membrane

Figure 13 shows that in the CBCT study, the "A" index prior to surgery was 6.37 mm in Group 1, 5.85 mm in Group 2, 7.14 mm in Group 3, and 7.15 mm in Group 4. The "A" index decreased from the starting values six months following regeneration therapy (Group 1 = 4.68 mm; Group 2 = 4.34 mm; Group 3 = 5.46 mm; Group 4 = 5.74 mm). Table 1 details the outcomes.

**Figure 14:**
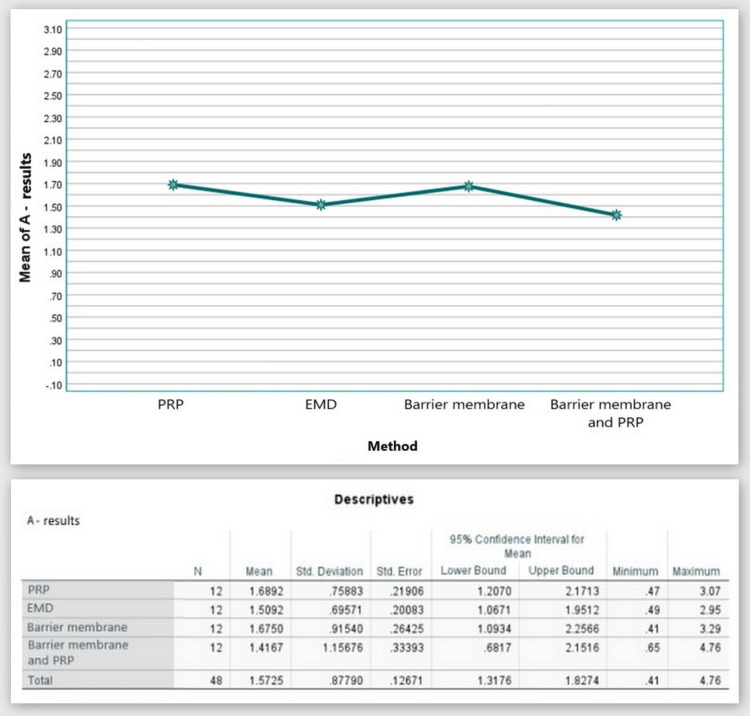
Statistical analysis results and graphics for parameter A

As visible in Figure 14, reduction in the distance from the CEJ to the base of the bone defect was observed in all four groups with mean values over six months of 1.69, 1.51, 1.68, and 2.14 mm, respectively.

The ANOVA test demonstrated that the findings for index “A” at six months post-regenerative therapy were not statistically significant among all four groups: F(3, 44) = 0.259, p = 0.854 > 0.05 (Appendix D).

В: the distance from the CEJ to the bone crest (on the CBCT)

**Figure 15:**
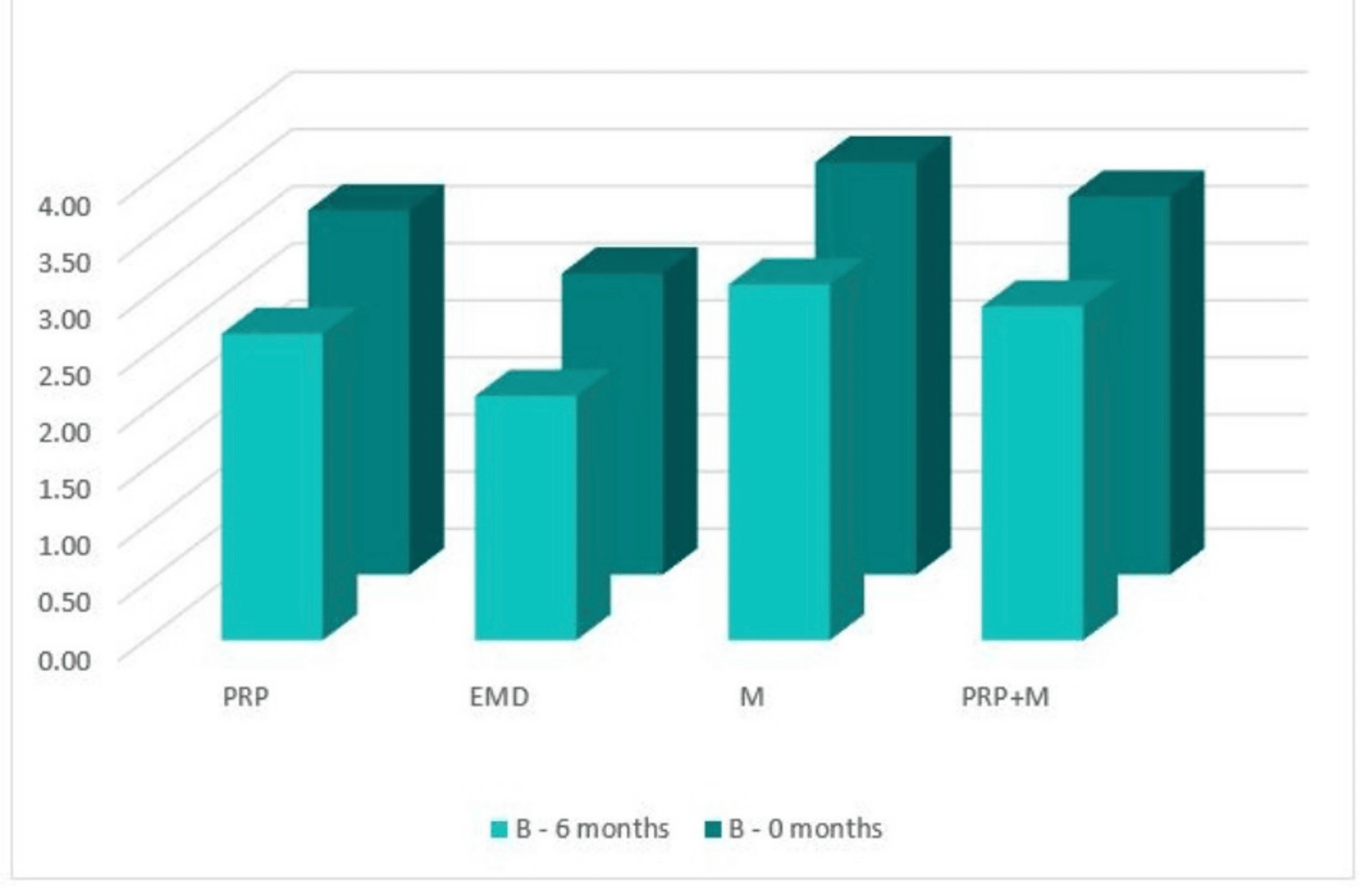
Parameter B comparison at 0 and six months PRP: platelet-rich plasma, EMD: enamel matrix derivative, M: barrier membrane, PRP+M: autogenous platelet-rich plasma and barrier membrane

Figure 15 shows that in the CBCT study, the "B" index prior to surgery was 3.19 mm in Group 1, 2.64 mm in Group 2, 3.61 mm in Group 3, and 3.31 mm in Group 4. The "B" index decreased from the starting values six months following regeneration therapy (Group 1 = 2.69 mm; Group 2 = 2.14 mm; Group 3 = 3.12 mm; and Group 4 = 2.93 mm). Table 1 details the outcomes.

**Figure 16:**
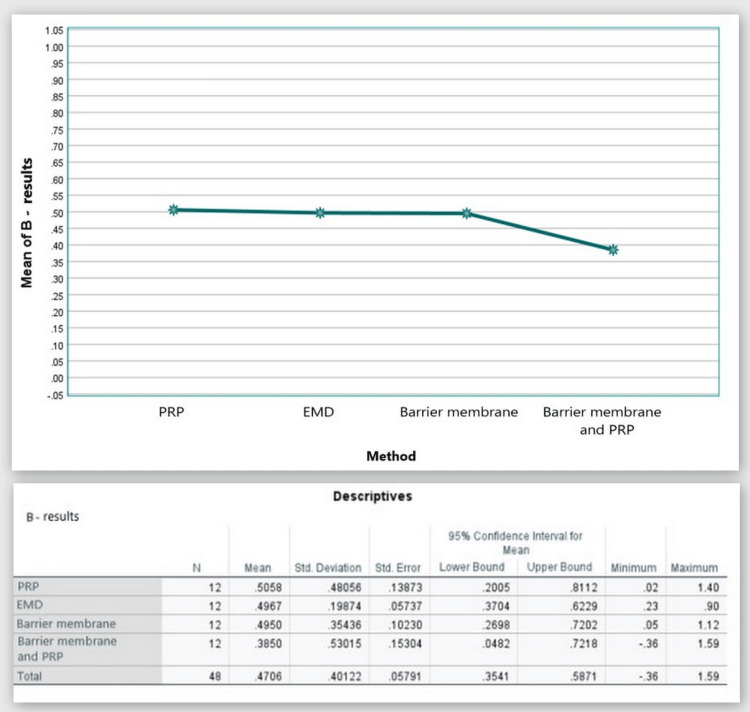
Statistical analysis results and graphics for parameter B

As visible in Figure 16, a reduction in the distance from the CEJ to the bone crest was observed in all four groups with mean values over six months of 0.51 mm, 0.50 mm, 0.50 mm, and 0.39 mm, respectively.

The ANOVA test demonstrated that the findings for index “B” at six months post-regenerative therapy were not statistically significant among all four groups: F(3, 44) = 0.233, p = 0.873 > 0.05 (Appendix E).

С: width of the bone defect (by CBCT)

**Figure 17:**
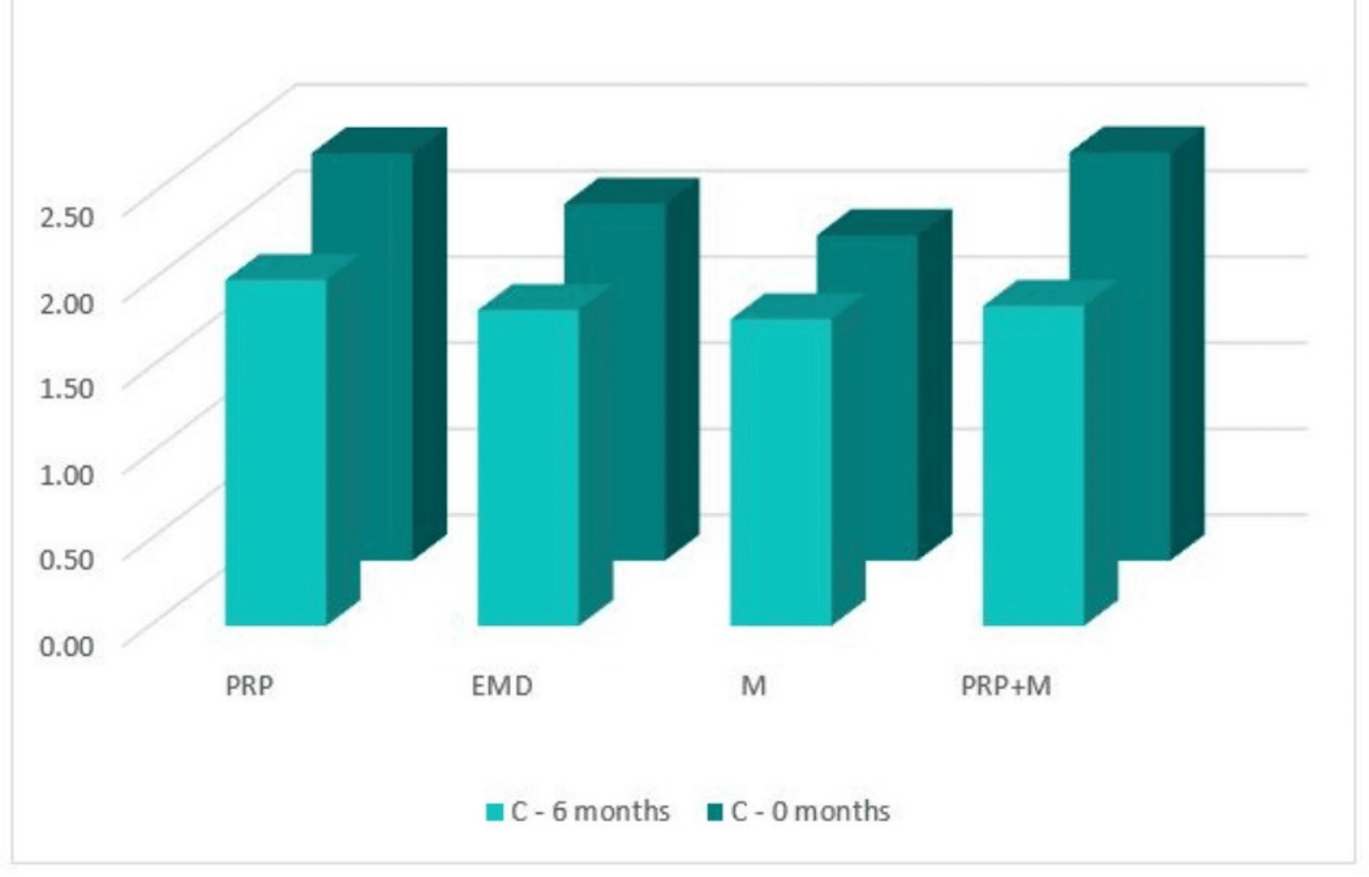
Parameter C comparison at 0 and six months PRP: platelet-rich plasma, EMD: enamel matrix derivative, M: barrier membrane, PRP+M: autogenous platelet-rich plasma and barrier membrane

Figure 17 shows that in the CBCT study, the "C" index prior to surgery was 2.37 mm in Group 1, 2.074 mm in Group 2, 1.89 mm in Group 3, and 2.37 mm in Group 4. The "C" index decreased from the starting values six months following regeneration therapy (Group 1 = 2.01 mm; Group 2 = 1.84 mm; Group 3 = 1.78 mm, and Group 4 = 1.86 mm). Table 1 details the outcomes.

**Figure 18:**
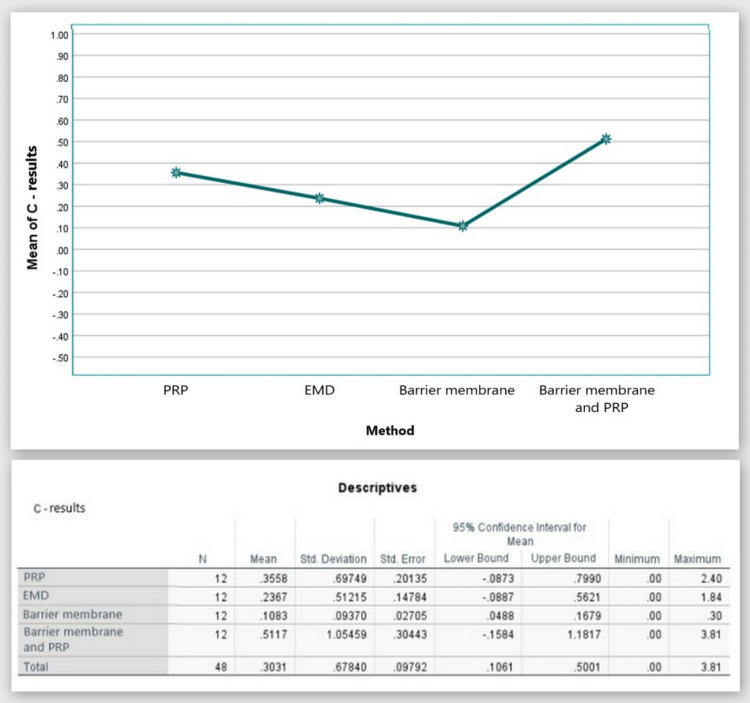
Statistical analysis results and graphics for parameter C

As visible in Figure 18, reduction in the width of the bone defect was observed in all four groups with mean values over six months of 0.36 mm, 0.24 mm, 0.11 mm, and 0.51 mm, respectively.

The ANOVA test demonstrated that the findings for index “C” at six months post-regenerative therapy were not statistically significant among all four groups: F(3, 44) = 0.758, p = 0.523 > 0.05 (Appendix F).

## Discussion

The prevalence of interproximal infraosseous defects was found to increase dramatically with age. In 2012, Eke et al. published their study assessing the incidence, severity, and broadness of periodontal disease in the US aged residents. It appears that periodontitis is a disease that occurs more frequently in men than in women [21]. In contrast to these data, in the present study, a greater number of patients were female.

In 2017, an epidemiological study estimated the prevalence of infraosseous defects in 329 adults by clinical and radiological examinations [22]. The study showed that IBDs predominate in the area of the second molars of the mandible. The outcomes of the present study corroborate those of Najim et al. (2017).

IBDs greatly raise the chance that the condition may worsen. A prompt and precise diagnosis is necessary for this. These days, CBCT is used to corroborate the clinical diagnosis of such problems. CBCT is the preferred imaging technique for capturing our results since it offers numerous benefits over two-dimensional X-ray examinations.

Regenerative therapy of periodontal IBDs results in decreased PPD and CAL gain, resulting in extended tooth life from five to 20 years with good supportive therapy [23,24,25].

The results of the clinical indicators "PPD" and "CAL" in this study are comparable to multiple studies conducted to date, demonstrating that regenerative therapy, regardless of the method used, results in a dramatic reduction in periodontal pocket probing depth and a significant gain in clinical attachment level [26,27,28,29,30].

Regarding the CBCT indicators, we have no basis for comparison at this stage, as these parameters have not been investigated so far.

Numerous surgical methods and materials have been developed over time to help regenerate periodontal bone deficiencies. In these days, the emphasis is on creating better biomaterials (barrier membranes, bone grafts, growth factors, and a combination of the aforementioned) to deliver even better outcomes in regenerative medicine [31,32,33]. This study validates the indisputable properties of some of the biomaterials that we often utilize in our clinic, such as barrier membranes and EMD.

The clinical and radiographic results of the four groups did not differ statistically significantly, according to our current investigation. We can therefore draw the conclusion that materials like PRP and EMD perform just as well as GTR. Here, it is important to make clear that the surgical procedure for using PRP and EMD in periodontal regenerative therapy is far simpler than that of GTR and that postoperative problems are also less common.

Limitations

As a major limitation of this study, it is important to note the characteristics of the bone defects (depth and width) as a possible factor influencing the results. Some of the bone defects were entirely two-walled, others were entirely three-walled, and still others were combined (two-walled and three-walled). This could somewhat influence the final results obtained.

Other limitations could be the short follow-up period of the present study and the rather limited sample of participants included in the study.

As guidelines for future studies, we can identify a larger sample of participants and a longer follow-up period.

## Conclusions

Regardless of the regenerative therapy technique used, all patients under examination showed improvements in imaging and clinical markers. The four patient groups' results did not differ in any way that was statistically significant.

Here, it is important to make clear that the surgical procedure for using PRP and EMD in periodontal regenerative therapy is far simpler than that of GTR, and that postoperative problems are also less common.

Concerning the methods of regenerative therapy with EMD and with the independent use of PRP, it should be noted that their surgical protocol is identical, but obtaining the PRP material is a harder procedure, more time-consuming and is associated with possible errors in methodology. The most significant advantage of the PRP method over the EMD method is the more affordable cost.

## Appendices

Appendix A

**Table 2:** ANOVA test with Tukey's HSD for PPD PPD: probing pocket depth, EMD: enamel matrix derivative, ANOVA: analysis of variance, HSD: honestly significant difference, PRP: platelet-rich plasma

Dependent variable	PPD	Mean			95% confidence interval	
(1) Method	(J) Method	Difference (I-J)	Std. error	Sig.	Lower bound	Upper bound
PRP	EMD	-667	.351	244	-1.60	.27
	Barrier membrane	-333	.351	779	-1.27	.60
	Barrier membrane and PRP	-417	.351	.639	-1.35	.52
EMD	PRP	667	.351	244	-.27	1.60
	Barrier membrane	.333	.351	779	-.60	1.27
	Barrier membrane and PRP	250	351	892	-.69	1.19
Barrier membrane	PRP	333	351	779	-.60	1.27
	EMD	-333	351	779	-1.27	.60
	Barrier membrane and PRP	-083	351	995	-1.02	85
Barrier membrane and PRP	PRP	417	351	639	-.52	1.35
	EMD	-250	351	892	-1.19	69
	Barrier membrane	.083	351	995	-.85	1.02

Appendix B

**Table 3:** ANOVA test with Tukey's HSD for GML GML: gingival margin level, EMD: enamel matrix derivative, ANOVA: analysis of variance, HSD: honestly significant difference, PRP: platelet-rich plasma

Dependent variable	GML	Mean			95% confidence interval	
(1) Method	(J) Method	Difference (I-J)	Std. Error	Sig.	Lower bound	Upper bound
PRP	EMD	583	.353	.360	-.36	1.52
	Barrier membrane	417	.353	.642	-.52	1.36
	Barrier membrane and PRP	-.167	.353	.965	-1.11	.77
EMD	PRP	-.583	.353	.360	-1.52	.36
	Barrier membrane	-.167	353	965	-1.11	.77
	Barrier membrane and PRP	-750	.353	.161	-1.69	.19
Barrier membrane	PRP	-417	353	642	-1.36	.52
	EMD	167	353	965	-77	1.11
	Barrier membrane and PRP	-,583	353	360	-1.52	36
Barrier membrane and PRP	PRP	167	353	965	-77	1.11
	EMD	750	353	.161	-19	1.69
	Barrier membrane	583	353	360	-.36	1.52

Appendix C

**Table 4:** ANOVA test with Tukey's HSD for CAL CAL: clinical attachment level, EMD: enamel matrix derivative, ANOVA: analysis of variance, HSD: honestly significant difference, PRP: platelet-rich plasma

Dependent variable	CAL	Mean			95% confidence interval	
(1) Method	(J) Method	Difference (I-J)	Std. Error	Sig.	Lower bound	Upper bound
PRP	EMD	-083	475	998	-1.35	1.19
	Barrier membrane	.083	475	998	-1.19	1.35
	Barrier membrane and PRP	-583	475	613	-1.85	.69
EMD	PRP	.083	475	998	-1.19	1.35
	Barrier membrane	.167	475	985	-1.10	1.44
	Barrier membrane and PRP	-.500	475	.720	-1.77	.77
Barrier membrane	PRP	-.083	.475	.998	-1.35	1.19
	EMD	-.167	.475	.985	-1.44	1.10
	Barrier membrane and PRP	-.667	.475	.504	-1.94	.60
Barrier membrane and PRP	PRP	583	.475	.613	-69	1.85
	EMD	.500	.475	.720	-77	1.77
	Barrier membrane	.667	475	.504	-.60	1.94

Appendix D

**Table 5:** ANOVA test with Tukey's HSD for Parameter A EMD: enamel matrix derivative, ANOVA: analysis of variance, HSD: honestly significant difference, PRP: platelet-rich plasma

Dependent variable	Parameter A	Mean			95% Confidence interval	
(1) Method	(J) Method	Difference (I-J)	Std. error	Sig.	Lower bound	Upper bound
PRP	EMD	.18000	36719	961	-8004	1.1604
	Barrier membrane	01417	36719	1.000	-9662	9946
	Barrier membrane and PRP	27250	36719	880	-7079	1.2529
EMD	PRP	-18000	36719	961	-1.1604	8004
	Barrier membrane	-16583	36719	969	-1.1462	8146
	Barrier membrane and PRP	09250	36719	994	-8879	1.0729
Barrier membrane	PRP	-01417	36719	1.000	-9946	9662
	EMD	.16583	36719	969	-8146	1.1462
	Barrier membrane and PRP	25833	36719	895	-7221	1.2387
Barrier membrane and PRP	PRP	-27250	36719	880	-1.2529	7079
	EMD	-09250	36719	994	-1.0729	8879
	Barrier membrane	-25833	36719	895	-1.2387	7221

Appendix E

**Table 6:** ANOVA test with Tukey's HSD for parameter B EMD: enamel matrix derivative, ANOVA: analysis of variance, HSD: honestly significant difference, PRP: platelet-rich plasma

Dependent variable	Parameter B	Mean			95% confidence interval	
(1) Method	(J) Method	Difference (I-J)	Std. error	Sig.	Lower bound	Upper bound
PRP	EMD	00917	16796	1.000	-4393	4576
	Barrier membrane	01083	.16796	1.000	-4376	4593
	Barrier membrane and PRP	12083	.16796	889	-3276	5693
EMD	PRP	-00917	.16796	1.000	-4576	4393
	Barrier membrane	00167	.16796	1.000	-4468	.4501
	Barrier membrane and PRP	11167	16796	910	-3368	5601
Barrier membrane	PRP	-01083	16796	1.000	-4593	4376
	EMD	-00167	16796	1.000	-4501	4468
	Barrier membrane and PRP	11000	16796	913	-3385	5585
Barrier membrane and PRP	PRP	-12083	16796	889	-5693	3276
	EMD	-11167	16796	910	-5601	3368
	Barrier membrane	-11000	16796	913	-5585	3385

Appendix F

**Table 7:** ANOVA test with Tukey's HSD for parameter C EMD: enamel matrix derivative, ANOVA: analysis of variance, HSD: honestly significant difference, PRP: platelet-rich plasma

Dependent variable	Parameter C	Mean			95% confidence interval	
(1) Method	(J) Method	Difference (I-J)	Std. error	Sig.	Lower bound	Upper bound
PRP	EMD	11917	27912	974	-6261	8644
	Barrier membrane	24750	27912	812	-4977	9927
	Barrier membrane and PRP	-15583	27912	944	-9011	5894
EMD	PRP	-11917	27912	974	-8644	6261
	Barrier membrane	12833	27912	967	-6169	8736
	Barrier membrane and PRP	-27500	27912	759	-1.0202	4702
Barrier membrane	PRP	-24750	27912	812	-9927	4977
	EMD	-12833	27912	967	-8736	6169
	Barrier membrane and PRP	-40333	27912	479	-1.1486	3419
Barrier membrane and PRP	PRP	15583	27912	944	-5894	9011
	EMD	27500	27912	759	-4702	1.0202
	Barrier membrane	40333	27912	479	-3419	1.1486
